# The Shape of the Olfactory Bulb Predicts Olfactory Function

**DOI:** 10.3390/brainsci12020128

**Published:** 2022-01-18

**Authors:** Xiaoguang Yan, Akshita Joshi, Yunpeng Zang, Francisca Assunção, Henrique M. Fernandes, Thomas Hummel

**Affiliations:** 1 Smell and Taste Clinic, Department of Otorhinolaryngology, TU Dresden, Fetscherstrasse 74, 01307 Dresden, Germany; joshiakshita93@gmail.com (A.J.); yunpengzang23@gmail.com (Y.Z.); thomas.hummel@tu-dresden.de (T.H.); 2 Flavour Institute, Department of Clinical Medicine, Aarhus University, Noerrebrogade 44, 1A, 8000 Aarhus, Denmark; francisca.s.assuncao@gmail.com (F.A.); henrique.fernandes@clin.au.dk (H.M.F.); 3 Center for Music in the Brain, Department of Clinical Medicine, Aarhus University, Noerrebrogade 44, 1A, 8000 Aarhus, Denmark; 4 Hedonia Research Group, Department of Psychiatry, University of Oxford, Oxford OX1 2JD, UK

**Keywords:** olfactory bulb shape, deformation, olfaction, plasticity, MRI, anosmia, smell

## Abstract

The olfactory bulb (OB) plays a key role in the processing of olfactory information. A large body of research has shown that OB volumes correlate with olfactory function, which provides diagnostic and prognostic information in olfactory dysfunction. Still, the potential value of the OB shape remains unclear. Based on our clinical experience we hypothesized that the shape of the OB predicts olfactory function, and that it is linked to olfactory loss, age, and gender. The aim of this study was to produce a classification of OB shape in the human brain, scalable to clinical and research applications. Results from patients with the five most frequent causes of olfactory dysfunction (*n* = 192) as well as age/gender-matched healthy controls (*n* = 77) were included. Olfactory function was examined in great detail using the extended “Sniffin’ Sticks” test. A high-resolution structural T2-weighted MRI scan was obtained for all. The planimetric contours (surface in mm^2^) of OB were delineated manually, and then all surfaces were added and multiplied to obtain the OB volume in mm^3^. OB shapes were outlined manually and characterized on a selected slice through the posterior coronal plane tangential to the eyeballs. We looked at OB shapes in terms of convexity and defined two patterns/seven categories based on OB contours: convex (olive, circle, and plano-convex) and non-convex (banana, irregular, plane, and scattered). Categorization of OB shapes is possible with a substantial inter-rater agreement (Cohen’s Kappa = 0.73). Our results suggested that non-convex OB patterns were significantly more often observed in patients than in controls. OB shapes were correlated with olfactory function in the whole group, independent of age, gender, and OB volume. OB shapes seemed to change with age in healthy subjects. Importantly, the results indicated that OB shapes were associated with certain causes of olfactory disorders, i.e., an irregular OB shape was significantly more often observed in post-traumatic olfactory loss. Our study provides evidence that the shape of the OB can be used as a biomarker for olfactory dysfunction.

## 1. Introduction

The olfactory bulb (OB) plays an important role in the processing of olfactory information. From an evolutionary perspective, the OB is one of the earliest structures of the vertebrate brain to develop [1,2]. The OB has also been suggested to serve as a repository of resident progenitor cells in the mature human brain, receiving neuroblasts migrating to it via a lateral ventricular extension [3,4]. It is widely accepted that odor coding is based on the spatiotemporal pattern of activation of the olfactory glomeruli in the OB [5,6].

OB has gained attention in the field of clinical research. Using magnetic resonance imaging (MRI), olfactory dysfunction can be gauged based on OB volumetrics [7,8]. Cutoff volumes separating normal volumes from hypoplastic ones have been established [9]. Results based on longitudinal studies showed how changes in OB volumes associate with improved olfactory function secondary to treatment [10]. Therefore, the assessment of OB structure is considered to provide diagnostic and prognostic information on a morphological basis. However, all the previous studies focused on the size and volume of the OB. They neglected the relation between OB shape and olfactory function.

To address this gap, the current study aimed (1) to explore potential differences in OB shapes between patients with the five most frequent etiologies of olfactory dysfunction and healthy controls and (2) to examine the relationship between OB shape and olfactory function, age, gender, and OB volume.

## 2. Methods

In this study, we used the data from datasets acquired within the context of six different studies. All these studies had been approved by the TU Dresden, Medical Faculty Ethics Review Board (EK96032015, EK348092018, EK56022016, EK262082010, EK203052017, EK 235072018). All subjects provided written informed consent before their detailed evaluation. A total of 192 patients (86 men) complaining of smell loss, with ages ranging from 24 to 81 years (mean ± standard deviation; 57 ± 12.8 years) and 77 healthy subjects (39 men), with ages ranging from 25 to 82 years (51 ± 15.5 years), were included in this cross-sectional, retrospective study. Patient groups consisted of subjects visiting the Smell and Taste Clinic of the Department of Otorhinolaryngology at TU Dresden starting from 2016 until 2020. Diagnosis of acquired olfactory dysfunction was made according to the recent Position Paper on Olfactory Dysfunction [11]. As part of their assessment, all patients underwent an evaluation that included an endoscopical examination of the nasal cavity, structured history collection, olfactory testing, and structural T2-weighted magnetic resonance imaging (MRI) [12]. Classification of the olfactory dysfunction was based on the underlying etiological conditions, i.e., olfactory dysfunction secondary to chronic rhinosinusitis (CRS), post-infectious olfactory dysfunction (PIOD), posttraumatic olfactory dysfunction (PTOD), olfactory dysfunction associated with neurological disease (patients with olfactory dysfunction related to Parkinson’s disease were recruited in this study), and idiopathic olfactory dysfunction. A high-resolution structural T2-weighted MRI scan was obtained for all. Patients with congenital anosmia were excluded based on their history and brain MRI examinations. All healthy subjects had no history of an underlying or preceding major medical condition and reported a normal sense of smell. Subjects’ demographic information and olfactory function are shown in Table 1.

### 2.1. MRI Acquisition

MRI acquisitions were performed on a 3T scanner (Siemens, Erlangen, Germany) with an 8-channel phase-array head coil. A standardized MRI for structural analyses was performed for all subjects, targeting structures that consisted of the left and right OB with a coronal T2-weighted fast spin-echo sequence: TR/TE = 4800/152 ms; slice thickness 2 mm; matrix size 256 × 256; 30 slices; averages 2; in-plane resolution 0.4 × 0.4 mm and no intersection gap) covering the anterior and middle segments of the base of the skull. 

### 2.2. Olfactory Testing

Quantitative testing of olfactory function was performed using the ‘‘Sniffin’ Sticks’’ test (Burghart GmbH, Wedel, Germany) [13]. Odorants were presented with pen-like devices. It was comprised of three tests, namely odor threshold with 16 increasing concentrations of phenyl ethyl alcohol using a single staircase, 3-alternative forced choice (3AFC) procedure (range of scores: 1 to 16). Odor discrimination was determined with a 3AFC procedure and 16 triplets (range of scores 0 to 16). Odor identification was tested using a 16-item multiple forced choice test (range of scores 0–16). The three subsets of this test were summated into the so-called “threshold-discrimination-identification (TDI) score”, leading to a maximum score of 48. For odor presentation, the pen’s cap was removed by the experimenter for approximately 3s and the felt tip was placed approximately 2 cm in front of the subjects’ nostrils. 

### 2.3. Evaluation of the Volume of the OB

We randomly chose 90 subjects (45 patients and 45 age- and sex-matched healthy control) from the cohort investigated here to evaluate the OB volume. The OB volume was measured on coronal T2-weighted MR images with ITK-SNAP software (Version 3.8.0, Pennsylvania, PA, USA). The planimetric contours (surface in mm^2^) of OB were delineated manually by an experienced observer blinded to the subjects. Then, all surfaces were added and multiplied to obtain the volume of OB segment in mm^3^. This approach of calculating OB volumes has been shown to be highly reliable and accurate [7,14,15].

### 2.4. Evaluation of the Shape of the OB

**Figure 1:**
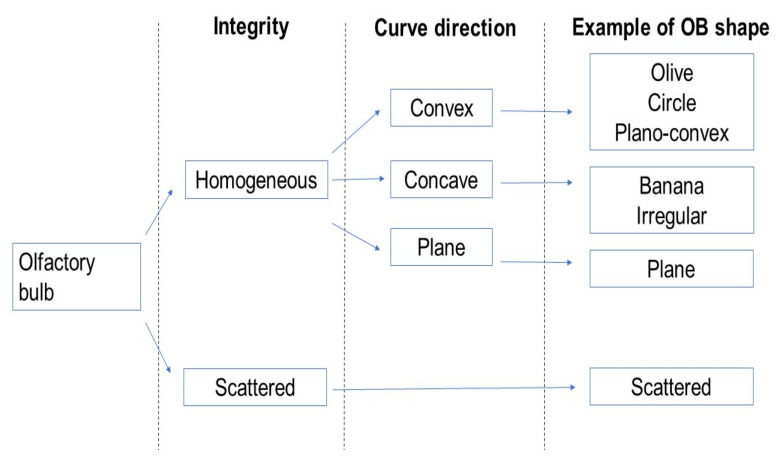
Classification of OB shapes.

**Figure 2:**
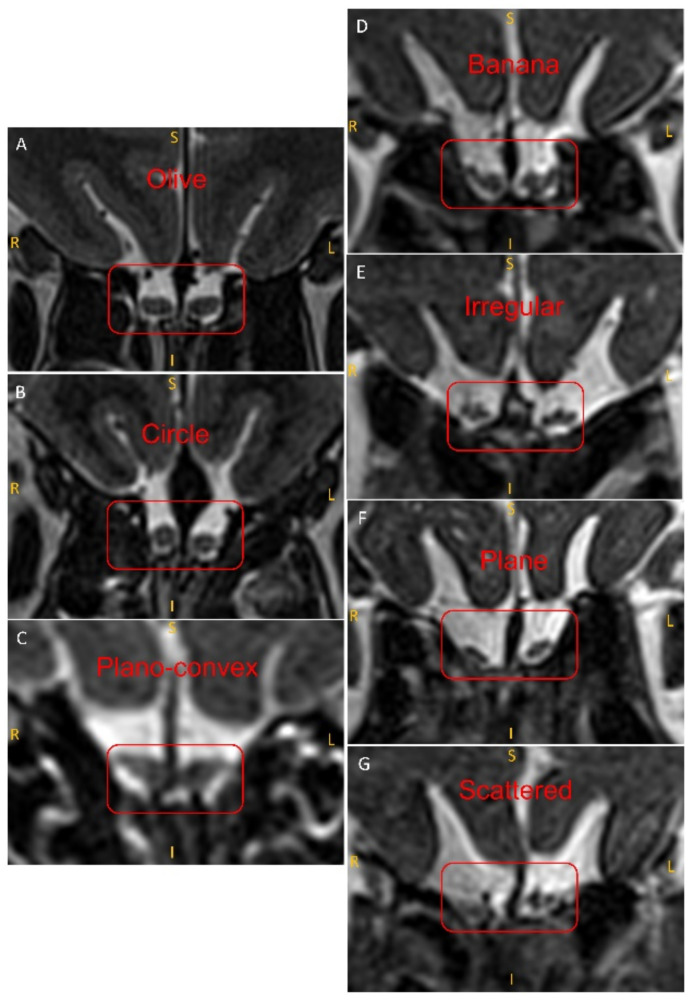
OB shapes are shown in the coronal T2-weighted MR image (red box) (R-Right, L-Left, S-Superior/Dorsal, I-Inferior/Ventral). (**A**) 23-year-old female healthy control (TDI = 37.25) with an olive-shaped OB; (**B**) 31-year-old healthy male control (TDI = 33.5) with circular OB; (**C**) 55-year-old woman with post-viral olfactory function (TDI = 12) demonstrating plano-convex OB; (**D**) 55-year-old man with post-viral olfactory function (TDI = 15) demonstrating banana-shaped OB; (**E**) 57-year-old woman with post-traumatic olfactory function (TDI = 23) showing irregular OB; (**F**) 54-year-old woman with post-viral olfactory function (TDI = 15) demonstrating plane OB; (**G**) 89-year-old woman with post-viral olfactory function (TDI = 20.5) demonstrating scattered OB. TDI = Threshold-Discrimination-Identification.

MRI scans were examined using ITK-SNAP software (Version 3.8.0, Pennsylvania, PA, USA), which allows images to be viewed in three orthogonal planes. Configurations of cross-sectional areas of OB were visually inspected in the slice through the most posterior coronal plane tangent to the eyeballs, in accordance to previous research [16]. Heuristically, based on opinions from a small group of experts, we chose to look at the shape of the OB according to its integrity and divided OBs into those homogeneous and scattered OBs. In homogeneous OB contours, the shape was considered by its outline as convex, concave, or plane. Convex OBs curve outward; concave OBs curve inward; and plane OBs are flat on both sides. The convex OB shape is then subdivided further into an olive shape, circle, or a plano-convex shape. For the concave OB shape, either the banana shape or an irregular shape is determined (Figure 1 and Figure 2) [17,18]. Images were assessed by a trained clinical expert (YZ) who was blinded to other clinical data of the respective cases. To assess inter-observer reliability, 107 cases were randomly analyzed by the observers (YZ and AJ). The inter-rater agreement between two independent persons analyzing the OB shape was substantial (Cohen’s Kappa = 0.73, 95% CI = 0.60–0.86, *p* < 0.001). 

### 2.5. Statistical Analysis

Seventy-seven patients were randomly selected and individually matched by age (within ± 2 years) and gender with the healthy group. OB shapes distribution were compared between patients and healthy controls using chi-squared tests. A chi-squared test was performed to compare the two OB shape patterns for gender differences. ANOVA and Student’s *t*-test were computed to examine the relation between OB shapes, age, and causes of smell loss. A non-parametric Mann–Whitney U test was applied to compare olfactory function among groups with different OB shape patterns. Multivariate linear regression analyses were performed using TDI, T, D, and I as dependent variables, with the shape of OB, age, and gender defining the set of independent variables. Moreover, differences for OB shapes, age, gender, and olfactory function were compared separately in patients and healthy controls. Statistics and graphical visualization were performed using GraphPad Prism 9.3.1 (GraphPad Software, Inc., La Jolla, CA, USA). A *p*-value threshold of 0.05 was used to define statistical significance. As the olfactory system tends to exhibit a right-sided dominance in processing, in our study statistical analyses were only performed based on the data from the right OB [19].

## 3. Results

### 3.1. Participant Demographics

A total of 192 patients with five different causes of olfactory dysfunction and 77 healthy controls were included in this study. Then, 77 patients were individually matched with the control group in term of age and gender. See Table 1 for the subjects’ demographic characteristics and olfactory function scores. We found no significant differences in age (t = 0.07, *p* > 0.05) and gender (χ^2^ = 0, *p* > 0.05) between cohorts with patients or healthy controls. As expected, significant differences were found in olfactory function between the patients and healthy controls (t = 13.7, *p* < 0.05).

### 3.2. Comparison of Patients and Controls in Terms of OB Shapes

**Figure 3:**
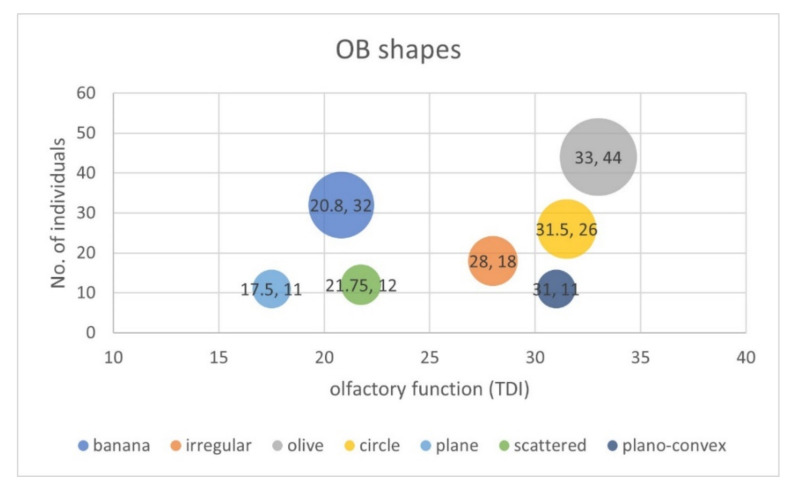
Bubble chart shows median TDI olfactory scores and number of investigated participants for OB shapes within each bubble (TDI scores; number). The size of the bubble indicates the number of individuals with the respective characteristic. Olive-shaped (28%, 44/154) and banana-shaped OBs (21%, 32/154) were the two most common types of OB shapes. Olive-shaped, circle, and plano-convex OB shapes had relatively higher median TDI total scores than other OB shapes. TDI = Threshold-Discrimination-Identification.

**Figure 4:**
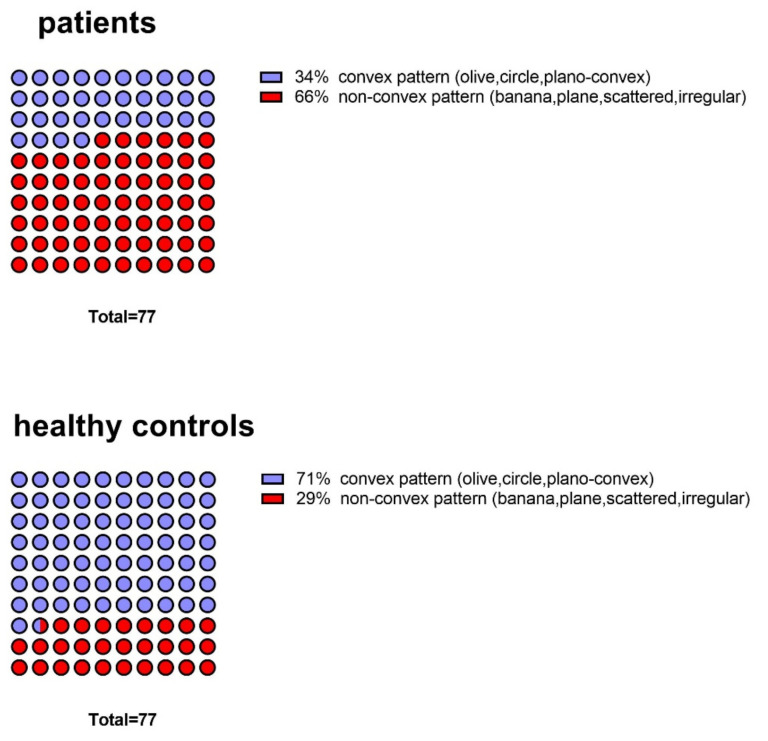
Pattern of OB shapes in patients and healthy controls.

Across matched groups (patients, *n* = 77; healthy control, *n* = 77), the data showed that olive-shaped (probability of occurrence (PO) = 28%) and banana-shaped OBs (PO = 21%) were the two most common types of OB shapes (Figure 3). Among patients, the banana shape (PO = 32%) was the most common shape, while in controls the olive-shaped (PO = 44%) was most frequent. The frequency of OBs with non-convex shape was significantly higher in patients with olfactory dysfunction (PO = 66%) than in healthy controls (PO = 29%) (χ^2^ = 21.9, *p* < 0.001) (Figure 4).

### 3.3. Correlation of OB Shapes with the Olfactory Function 

**Figure 5:**
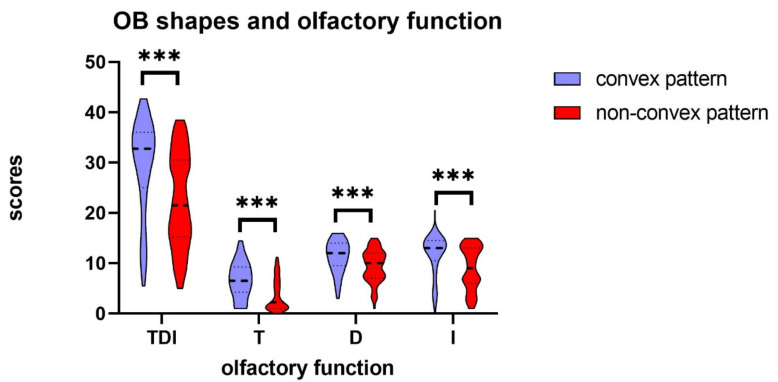
Violin plot comparisons of olfactory scores between subjects with convex pattern OB and subjects with non-convex pattern OB showing the median (a black dash line in the center of violin), and interquartile range (the black dot line on the violin plot). Subjects with non-convex pattern OB had significantly lower TDI total scores and T, D, and I component scores compared to subjects with convex pattern OB (all *P*’s < 0.001), *** *p* < 0.001. TDI = Threshold-Discrimination-Identification. T = odor threshold; D = odor discrimination; I = odor identification.

Our results indicated an association between specific OB shapes and olfactory function. Across matched subjects, olive-shaped OB had the highest median TDI score (TDI = 33) (Figure 3). The plane OB shape had the lowest median TDI score (TDI = 17.5) (Figure 3). More interestingly, our results suggest that the TDI scores of the convex OB patterns, i.e., olive, circle, and plano-convex OBs, were associated with relatively higher olfactory scores than the other OB types. Statistical analysis showed that the non-convex OB pattern, in comparison to the convex OB pattern, came with a significantly lower olfactory function, defined by olfactory threshold, odor discrimination, odor identification, and the composite olfactory test score (U = 1644.50, U = 1967.50, U = 1869.00, and U = 1703.50, respectively; *p* < 0.001) (Table 2 and Figure 5). Results from multiple regression analyses showed that differences in the shape of the OB, observed in relation to olfactory function, was independent of age, gender, and OB volume (*p* < 0.05). Multiple logistic regression adjusted for age, gender, and OB volume indicated an association with olfactory dysfunction for OB shape (odds ratio [OR] convex vs non-convex, 4.5 [95% CI, 2.0–6.5]).

Neither in the group of patients nor in the group of healthy controls did we observe significant differences in the TDI score between convex and non-convex OB pattern groups (patients: t = 0.69, *p* = 0.49, controls: t = 1.91, *p* = 0.06).

### 3.4. OB Shape and OB Volume

Compared with subjects with non-convex OB shape, subjects with convex OB shape had a significantly higher OB volume (47.0 ± 16.0 mm^3^ vs. 37.8 ± 18.3 mm^3^, *p* < 0.05). There were no significant differences among the seven types of OB shape and OB volume (F = 1.77, *p* > 0.05).

### 3.5. Correlation of OB Shape with Age 

Across matched subjects, there was no significant difference in age between the convex and non-convex OB pattern group (t = 0.10, *p* = 0.92).

Subjects with scattered a OB shape had the highest average age (75 ± 9.1 years, *n* = 3) compared to other OB shapes (olive: 48 ± 13.9 years, *n* = 33; circle: 55 ± 14.8 years, *n* = 16; triangle: 43 ± 15.2 years, *n* = 6; banana: 55 ± 14.7 years, *n* = 8; strip: 42 ± 3.8 years, *n* = 3; irregular:51 ± 21.7 years, *n* = 8). Indeed, the frequency of non-convex OB patterns was significantly higher in the group of older healthy subjects (age > 60 years, *n* = 21, 9/21 = 43%) compared to younger subjects (age ≤ 60 years, *n* = 56, 11/56 = 20%) (χ^2^ = 4.28, *p* < 0.05). However, the mean age of subjects with a convex pattern (50 ± 14.3 years, *n* = 57) and a non-convex pattern (54 ± 18.6 years, *n* = 20) was statistically not different between groups (t = 1.17, *p* = 0.25).

### 3.6. OB Shapes Associated with the Causes of Olfactory Loss in Patients

According to the causes of smell impairment, patients were categorized into five groups. Group 1 (G1) consisted of 57 patients with an idiopathic smell disorder. Group 2 (G2) contained 31 patients with smell impairment second to CRS, group 3 (G3) 58 patients with postinfectious olfactory loss, group 4 (G4) 45 patients with post traumatic olfactory loss, and group 5 (G5) with 11 patients with idiopathic Parkinson syndrome. Prevalence of OB shapes in patients with different causes were shown (Table 3). Additionally, we found that an irregular OB shape was significantly more often observed in post-traumatic olfactory loss (*n* = 45, 12/45 = 26.7%) than in other etiologies of olfactory loss (idiopathic: *n* = 57, 9/57 = 15.8%, χ^2^ = 1.82; postinfectious: *n* = 58, 9/58 = 15.5%, χ^2^ = 1.94; CRS: *n* = 21, 3/21 = 14.3%, χ^2^ = 1.25; *p* < 0.05). There is no significant difference for the number of patients with convex and those with non-convex patterns among different causes of olfactory dysfunction (χ^2^ = 5.5, *p* = 0.237).

## 4. Discussion

Our study proposes a new framework to classify the shape of the human OB and provides first evidence of the association between OB shape and olfactory dysfunction, using a large sample size of healthy controls and patients with different types of olfactory dysfunction. Our observations indicate the presence of morphological changes in the OB, from regular to irregular and from integrity to disintegration. These changes are linked to human olfactory function, even when adjusting for age, gender, and OB volume. In addition, we found that the OB exhibits increased deformation with age across healthy subjects. Furthermore, we found that an irregular OB shape was significantly more often observed in post-traumatic olfactory loss. 

In addition to the typical olive-shaped OB, which reflects a normal, healthy status of the OB, various other OB shapes were also observed in this study, for example, the banana-shaped OB with the convexity of the walls. These forms are common in patients with smell deficits and are also found in healthy subjects. Such an overlap in OB shapes between patients and controls could be at least partly explained by the fact that some controls may have olfactory dysfunction without being aware of it [20,21]. Consistent with this, previous studies have described the variability of the OB structure, and especially the OB volume. Buschhüter et al. [9] reported that OB volumes decrease with age in healthy people. They proposed normative data of OB volume in relation to age based on results from 125 patients. Furthermore, a decrease of OB volume has also been reported in patients with olfactory dysfunction due to various causes [14,15,16,22]. Moreover, in a longitudinal study, Gudziol et al. [10] described an OB change post treatment in 19 CRS patients, which was correlated with olfactory function. Another study, following a period of 4 months of olfactory training, showed that 97 healthy people exhibited an average increase of OB volume [23].

At a macroscopic level, Burmeister et al. [18] depicted laminar patterns of the human OBs (*n* = 24). They identified three layers in 8.3%, two layers in 83.3%, and one layer in 8.3% of their subjects using high resolution 3T MRI. At a cellular level, the spatial distribution of glomeruli in the human OB is irregular and complex [24] and shows higher variability than what is seen in animals [25,26].

The structural variability in OB shapes and volumes appears to be an expression of OB plasticity. As the OB receives olfactory input from the olfactory epithelium, it is natural to think that OB structure is altered secondary to reduced sensory input. In fact, OB volume has been observed to be associated with nasal septal deviation, with OB volume being significantly lower at the narrower side, which may indicate a “bottom-up modulation” of the OB [27]. Importantly, the OB also appears to be subject to “top-down modulation”, showing in some neurodegenerative diseases as well as mental disorders, which are associated with a smaller OB volume [28,29,30]. Top-down modulation also became evident in an experiment on lateralized olfactory exposure (“olfactory training”) in healthy subjects who exhibited an increased OB volume not just on the side exposed to odors but on the ipsi- and contralateral sides [31].

OB plasticity may depend on numerous factors that are currently discussed, for example, (1) continuous neuronal supply from the subventricular zone (SVZ), where young neurons migrate within the rostral migratory stream and replace interneurons (periglomerular cells and granular cells) in the OB [3]; (2) continuous synaptogenesis with dendrites of mitral/tufted cells occurring from incoming axonal projections of olfactory receptor neurons at the glomerular level; and (3) in a recent animal study, a new form of structural remodeling of adult-born OB neurons suggest direct neurogenesis within the OB itself [32]. Emerging evidence for OB plasticity indicates that OB is not just a static relay station, but dynamically processes olfactory information based on experience and context [33].

Considering that pronounced morphological changes of the olfactory epithelium are found with anosmia or aging, and that information regarding individual odorant processed by olfactory sensory neurons (OSNs) in the olfactory epithelium (OE) is integrated into distinct subsets of glomeruli in a stereotyped region of the OB, we hypothesize that changes of patterns at the level of the OE may induce specific changes in OB shape [34,35,36].

The association between shape changes of sensory related regions and function have been reported, for example for the auditory system or the visual systems [37,38]. For the olfactory system, findings from the present study are in line with prior evidence suggesting that the OB structure is associated with olfactory function. Such correlation has been shown not only for overall olfactory ability, but also for the subcomponent’s odor identification and odor threshold [15,39,40]. Even dynamic changes in odor threshold were significantly and positively correlated with changes in OB volume [10]. However, the volumetric evaluation of OB is not yet a routine procedure for patients with olfactory dysfunction in a typical outpatient clinic, since it requires special software and some time needed for OB segmentation. Hence, our study presents an easier, simpler method to visually classify and assess OB deformation, with a large potential for clinical application as a biomarker for patients with olfactory dysfunction. For example, our study shows that a “plane” and “scattered” OB shape is generally associated with decreased olfactory function. 

To note, the present results suggested that OB morphology and olfactory function correlate, but that a reliable individual diagnosis based on OB morphology alone is not possible. Although the group differences are clear, any one OB shape can be found in both patients and healthy controls. This overlap can partly explain that the banana OB shape is associated with lower olfactory function than what is found in subjects with an irregular OB shape, although both are concave shapes. Specifically, the irregular OB shape (44%, 8/18) is more often found in healthy controls than is the banana OB shape (25%, 8/18). 

A clinically relevant finding was that we provided a new perspective to look at the OB shape in terms of the convexity—either a convex pattern or non-convex pattern. Non-convex OB patterns were significantly more often observed in patients than in controls. Therefore, the convexity of OB shape appears to reflect pathological changes of patients with olfaction dysfunction and can be a useful additional morphological parameter in the assessment of olfactory function. 

The plano-convex OB shape was attributed to the convex OB shape pattern. Even if this subgroup is classified as a non-convex pattern, the difference between patients and healthy controls is still present in terms of the ratio between convex and non-convex patterns (in patients, convex:non-convex = 27:73; in healthy controls, convex:non-convex = 64:36, χ^2^ = 27.6, *p* < 0.001).

We found differences in age-related OB shape in healthy participants. In humans, an aged OB shows a decrease of the volume of the OB, the concentration of mitral cells per unit area, and both layer thickness and the number of glomeruli [41,42]. However, the results in rodents are more complex. In mice, OB volume does not change [43], or may even increase with age [44]. In the present study, the subjects with scattered OB shape were the oldest, suggesting an age-related degeneration of OB structures. It is unclear whether this change in OB shape is related, for example, to the OB microstructure or the turnover of interneurons. Like other sensory systems, olfactory function decreases with increasing age, due to numerous reasons [45].

Also, in the current study, non-convex OB patterns occurred in the five most common etiologies for olfactory dysfunction with a high percentage. The highest prevalence of the irregular OB shapes was observed in post-traumatic olfactory dysfunction. This may result from the specific mechanisms, with a traumatic injury leading to the direct, sudden disruption of olfactory pathways, for example, direct shearing of the olfactory nerves and focal contusion within the OB. Of note, the olfactory system is among the earliest affected structures in neurodegenerative disease, such as Alzheimer’s disease and Parkinson’s disease. In the present study, the prevalence of non-convex OB shapes pattern was higher for subjects with neurodegenerative diseases in comparison to healthy controls.

Results from the current study support the idea of an association between OB shapes and olfactory function. Our findings raise numerous questions that future studies should address, namely (1) whether OB shapes can be complementary to volumetric assessment of longitudinal changes, (2) whether OB shapes are helpful in terms of prognostic information in patients with olfactory dysfunction, and (3) whether, and if so, how treatment induces changes in OB morphology. Overall, in this study, we visually classified and qualitatively assessed OB shapes, which is easy and simple in order to screen OB deformations. We deliberately pursued this path in order to provide a simple clinical metric to obtain more detailed information on olfactory dysfunction. Still, the current results suggest that OB morphology contains information on olfactory function, and possibly also on the prognosis of the disorder. Further research should provide more objective measures to characterize the OB shape and increase the inter-rater agreement.

## 5. Conclusions

The variability of OB shape was examined in humans. The categorization of OB shapes is possible with a good inter-rater agreement. Non-convex OB patterns were significantly more often observed in patients than in controls. Additionally, there is an association between OB shape and olfactory function. In healthy subjects, OB shape seemed to change with age. The results also indicated that OB shapes were associated with certain causes of olfactory disorders, i.e., an irregular OB shape was significantly more often observed in post-traumatic olfactory loss. 

## Figures and Tables

**Table 1:** Demographic characteristics of patients, results from olfactory testing.

Characteristic	Before Matching	After Matching			
	Patients	Patients	Healthy Control	Statistics	*p*
Age (mean [SD])	57 [12.8]	51 [15.2]	51 [15.5]	*t* = 0.07	NS
Gender (number (%))				χ^2^ = 0	NS
Male (*n* (%))	86 (45)	39 (49)	39 (49)		
Female (*n* (%))	106 (55)	38 (51)	38 (51)		
Total (*n*)	192	77	77		
Smell function (mean [SD])					
TDI	18.1 [7.6]	18.6 [8.5]	33.5 [4.2]	*t* = 13.7	*p <* 0.001
T	2.7 [2.7]	3.2 [3.1]	7.4 [2.9]	*t* = 8.6	*p <* 0.001
D	8.2 [3.0]	8.4 [3.0]	12.5 [2.1]	*t* = 10.2	*p <* 0.001
I	7.4 [3.5]	7.4 [4.1]	13.6 [1.5]	*t* = 12.3	*p <* 0.001

TDI = Threshold-Discrimination-Identification; T = odor threshold; D = odor discrimination; I = odor identification; NS = not significant; SD = standard deviation.

**Table 2:** Comparison between convex pattern and non-convex pattern across matched groups.

Characteristic	Convex Pattern	Non-Convex Pattern	Statistic	*p*
Age (mean [SD])	51 [15.2]	51 [15.5]	*t* = 0.106	NS
Gender, *n* (%)			χ^2^ = 0.922	NS
Male (*n* (%))	44 (54)	34 (47)		
Female (*n* (%))	37 (46)	39 (53)		
Total (*n*)	81	73		
Smell function (median)				
TDI	32.8	21.5	U = 1703.500	*p* < 0.001
T	6.5	2.3	U = 1644.500	*p* < 0.001
D	12	10	U = 1967.500	*p* < 0.001
I	13	9	U = 1869.000	*p* < 0.001

NS = not significant; TDI = Threshold-Discrimination-Identification; T = odor threshold; D = odor discrimination; I = odor identification; SD = standard deviation.

**Table 3:** Prevalence of OB shapes in patients with different causes.

Causes, OB Shape	Olive, no. (%)	Circle, no. (%)	Plano-Convex, no. (%)	Banana, no. (%)	Irregular, no. (%)	Plane, no. (%)	Scattered, no. (%)	Total
Sinonasal	4 (19.0%)	3 (14.3%)	2 (9.5%)	6 (28.6%)	3 (14.3%)	1 (4.8%)	2 (4.8%)	21
idiopathic	6 (10.5%)	5 (8.8%)	1 (1.8%)	18 (31.6%)	9 (15.8%)	4 (7.0%)	14 (24.6%)	57
Parkinson’s disease	1 (9.1%)	4 (36.4%)	0 (0.0%)	3 (27.3%)	3 (27.3%)	0 (0.0%)	0 (0.0%)	11
PIOD	3 (5.2%)	9 (15.5%)	7 (12.1%)	15 (25.9%)	9 (15.5%)	11 (19.0%)	7 (12.1%)	58
PTOD	9 (20.0%)	0 (0.0%)	4 (8.9%)	11 (24.4%)	12 (26.7%)	3 (6.7%)	4 (8.9%)	45
Total, no.	53	36	23	21	19	26	14	192

PIOD = post-infectious olfactory dysfunction, PTOD = posttraumatic olfactory dysfunction.

## Data Availability

The data that support the findings of this study are available on request from the corresponding author (Xiaoguang YAN, M.D., Smell and Taste Clinic, Department of Otorhinolaryngology, TU Dresden, Fetscherstrasse 74, 01307 Dresden, Germany; Tel.: +49-351-458-4189; Fax: +49-351-458-4326; xiaoguang.yan@tu-dresden.de). The data are not publicly available due to their containing information that could compromise the privacy of research participants.
